# Tensions in learning professional identities – nursing students’ narratives and participation in practical skills during their clinical practice: an ethnographic study

**DOI:** 10.1186/s12912-017-0238-y

**Published:** 2017-08-16

**Authors:** Mona Ewertsson, Sangeeta Bagga-Gupta, Renée Allvin, Karin Blomberg

**Affiliations:** 1 Faculty of Medicine and Health, School of Health Sciences, Örebro University, S-70182 Örebro, Sweden; 2 School of Education and Communication, Jönköping University, Jönköping, Sweden; 3 Clinical Skills Centre, Örebro University Hospital, Örebro, Sweden

**Keywords:** Clinical practice, Learning, Nursing students, Practical skills, Socialization

## Abstract

**Background:**

Clinical practice is a pivotal part of nursing education. It provides students with the opportunity to put the knowledge and skills they have acquired from lectures into practice with real patients, under the guidance of registered nurses. Clinical experience is also essential for shaping the nursing students’ identity as future professional nurses. There is a lack of knowledge and understanding of the ways in which students learn practical skills and apply knowledge within and across different contexts, i.e. how they apply clinical skills, learnt in the laboratory in university settings, in the clinical setting. The aim of this study was therefore to explore how nursing students describe, and use, their prior experiences related to practical skills during their clinical practice.

**Methods:**

An ethnographic case study design was used. Fieldwork included participant observations (82 h), informal conversations, and interviews (*n* = 7) that were conducted during nursing students’ (*n* = 17) clinical practice at an emergency department at a university hospital in Sweden.

**Results:**

The overarching theme identified was “Learning about professional identities with respect to situated power”. This encompasses tensions in students’ learning when they are socialized into practical skills in the nursing profession. This overarching theme consists of three sub-themes: “Embodied knowledge”, “Divergent ways of assessing and evaluating knowledge” and “Balancing approaches”.

**Conclusions:**

Nursing students do not automatically possess the ability to transfer knowledge from one setting to another; rather, their development is shaped by their experiences and interactions with others when they meet real patients. The study revealed different ways in which students navigated tensions related to power differentials. Reflecting on actions is a prerequisite for developing and learning practical skills and professional identities. This highlights the importance of both educators’ and the preceptors’ roles for socializing students in this process.

## Background

A nurse’s daily clinical work includes performing several different practical skills. In order to provide adequate care, maintain patient safety, and feel comfortable in the profession, registered nurses need to be equipped with requisite skills. Nursing students are required to learn such skills in preparation for their work as nurses [1]. However, there is a lack of knowledge and understanding regarding the ways in which nursing students learn practical skills and how they apply knowledge acquired in the clinical skills laboratory (CSL) in university settings and clinical practice within and across different contexts.

Performing a practical skill is a complex action that requires theoretical knowledge and a critical attitude adapted to each unique patient and specific context, based on current scientific evidence [2, 3]. This can be viewed as a challenge, especially in light of rapid technological developments which require nurses to be up to date with advancements in the field of nursing sciences [4]. Therefore, it is important that the curricula in nursing education promote opportunities for students to develop knowledge and skills in this respect. In other words, frequent opportunities to perform practical skills are needed. Likewise, there exists a need for a reflective academic approach and an understanding that research and evidence are necessary and cannot be separated from the process of learning practical skills [4–6].

Education for nursing varies from country to country. In Sweden the nursing programme lasts 3 years, leading to a bachelor’s degree. The programme was designed in response to a directive from the European Union that stipulated that at least one-third of the nursing education should consist of theoretical courses, with clinical education comprising a minimum of 50%. Part of the clinical training can be provided in the CSL [7, 8]. The CSL is today seen as an important learning arena that allows nursing students to learn and practise practical skills in a safe way, through simulation at different levels [9]. Previous studies show that students report that training in the CSL helps prepare them for, and gives them self-confidence prior to, practising in clinical settings [1, 10]. It also reduces some students’ concerns about managing practical skills in encounters with patients in real-life situations.

Education in clinical settings is a pivotal part of nursing education. A practicum in different clinical settings provides students with the opportunity to put the knowledge, skills and concepts they have acquired from lectures into practice with real patients, under the guidance of nurses [11, 12]. Furthermore, clinical experience is essential for shaping nursing students’ identity as future nurses. It is widely known that nurses who precept nursing students play a key role in the students’ socialization process; they also have a strong influence on the students’ learning process [13–16]. Students’ preparation for clinical practice facilitates the socialization process, which in turn facilitates their learning process: thus, a progression of students’ knowledge can be expected to occur [17]. What is taught to the students, what they do or see in clinical settings, and what they experience throughout their university education is what shapes their idea of what a professional nurse is and does [18].

Using a sociocultural framework for socialization and communication as a point of departure means that learning is seen as a social process that is a part of everyday practices. Learning is contextual and includes a dimension of participation in social practices in which cultural tools such as language and material tools and the use of artefacts shape students’ learning [19–21]. Hence, the practical skills nursing students learn and how they use them are viewed as dependent on the cultural and social contexts that they are part of. The fact that nursing students are provided with opportunities to learn practical skills in two different social settings, the CSL and the clinical setting, suggests that the need exists to facilitate a transfer of knowledge between these settings. Knowledge transfer is a complex process and – for the purposes of the present study – we define it as an application of knowledge from one specific situation to another [22]. A key factor in facilitating this process of knowledge transfer is that the learner can identify similarities across tasks and contexts. Furthermore, nursing education requires guiding students to see similarities and differences in learning situations so that they can understand skills in a wider perspective.

Several studies report that students feel satisfied with their learning in the CSL and feel prepared to perform practical skills; however, this does not automatically mean that the students have adequate knowledge and the capacity to transfer their knowledge from the CSL to clinical settings [23, 24].

The study presented here takes its point of departure in the fact that there is a gap in the research literature regarding the ways in which nursing students deploy CSL-acquired knowledge in clinical settings. Therefore, the aim of this study was to explore how nursing students describe, and use, their prior experiences related to practical skills during their clinical practice.

## Method

### Design

An ethnographic case study design, guided by a sociocultural perspective on learning and communication [20], was used. Ethnographically inspired methods are appropriate when *the objective is to* obtain in-depth understanding of the meanings, functions and practices of a group in a specific sociocultural context [25]. The focus was to explore social practices and everyday interactions in an authentic clinical setting where nursing students were placed for their clinical practice, and where they had possibilities to learn practical skills. Fieldwork included participant observations, informal conversations, and interviews conducted during the nursing students’ clinical practice.

Situations in which the students received opportunities to learn and be socialized into practical skills were viewed as “cases”. A case began when the preceptor and the nursing student were informed that they were in charge of a specific patient. This situation could entail practical skills and/or care actions performed in relation to the specific patient. The case was considered completed as soon as the preceptor and the student finished the assignment related to the specific patient and/or when they received information regarding a new patient. In some instances in our data, the preceptor and the nursing student were in charge of several patients simultaneously. Therefore, we have taken the stance that a case was completed when our observations and/or the actions related to an individual patient were completed.

### Settings and participants

The fieldwork was conducted in 2015 at an emergency department (ED) at a university hospital in Sweden. We chose this clinical setting because of the specific nature of the work in an ED. A range of patient care activities is provided in the ED setting and a comprehensive part of the nurse’s work is to perform a variety of practical skills. This type of setting provides nursing students with a range of opportunities where they can learn and develop practical skills of different kinds. This strategic choice potentially enabled rich descriptions related to the aim of the study [25]. A clinical teacher and a manager facilitated access to the ED. We included nursing students from a 3-year nursing programme at a university in Sweden. This programme leads to a bachelor’s degree and follows regulations set by the Swedish government and the Swedish Higher Education Authority. The students have 2-week clinical practice placements at the ED during their third or fourth semester.

All students at the included university hospital who had planned to do their clinical practice at the ED (*n* = 44) were invited to participate in this study. They received both written and verbal information about the study. Seventeen nursing students between 20 and 36 years of age (3 men and 14 women) agreed to participate. They were assigned a personal preceptor, but because of organizational factors they sometimes had to follow other nurses. The preceptors had varying experiences of precepting nursing students, ranging from it being their first time to having more than 20 years’ experience as preceptors.

### Data collection

Time spent on data collection during the fieldwork period was guided by a selective intermittent time mode; this entailed that observations were carried out until saturation was reached and recurring interactional patterns had been identified [26]. The fieldwork spanned two semesters and included day, evening and night shifts, resulting in a total of 82 h of participant observation. The majority of the fieldwork was conducted by the first author (ME); however, the second (SBG) and last author (KB) also participated in the fieldwork. Three of the four authors of this study have a range of educational and working experience in nursing and nursing-related work and have worked both in clinics and in university nursing education. The second author (SBG) has extensive experience of ethnographic design and fieldwork in the areas of learning and communication in different institutional settings. The researchers’ different expertise enabled a rich, analytically driven discussion on the culture of clinical practice. During fieldwork, the researchers strived to keep to their role of researchers and avoided participating in any precepting situations. They took field notes on learning situations where preceptors and students interacted, detailing what happened, in what context, what was said, and who was involved. Reports on informal conversations with preceptors and nursing students were also included in the field notes [25]. Furthermore, three situations were video-recorded, and notes on use of artefacts, and digital photos of the tools, instructions, wall charts, etc. at the ED were added to the fieldwork data. The researchers subsequently wrote personal reflections based upon their field notes and experiences in the field; these personal reflections were also taken into account in the analysis.

Guided by the findings from the fieldwork, individual interviews were conducted by the first author (ME). An interview guide with question areas was developed, based on what the students focused on when planning and performing practical skills, and how they acted when something they participated in or witnessed differed from the literature and the clinical practice guidelines. Seven students who had a clinical placement at the ED during our fieldwork were invited to also participate in an interview. All accepted and provided written consent before the interviews were conducted. The semi-structured interviews were audio-recorded and lasted between 25 and 35 min, and took place at locations chosen by the students. All interviews were transcribed verbatim.

### Data analysis

Following an ethnographic approach, a parallel process of data collection and analysis took place [25]. After each day at the ED, field notes were elaborated, and analytical memos were written by each of the researchers. Here, nursing students’ and preceptors’ activities related to practical skills were in focus. An overview of the data was created by reading the field notes several times with the aim of creating a holistic understanding of the data. All authors, including the third author (RA) who did not participate in the fieldwork, were involved in this process, individually as well as together. The researchers also worked in pairs to analyse the films and digital photos taken. Based on this first level of analysis, preliminary themes were identified, which then formed the basis for further data analysis [25].

The transcribed interviews were read and reread in detail by all researchers individually as well as in pairs. The themes that emerged were finally discussed by the research team; the intention was to identify overarching analytical issues regarding the nursing students’ descriptions of their experiences related to practical skills and how these descriptions related to their learning processes at the ED. After reading the interview transcripts several times, the authors coded and then sorted the data into preliminary themes. As a final step, the themes that emerged from the different data sets – field notes, video recordings and transcribed interviews – were compared and discussed by the team in order to arrive at an overarching theme, thus enhancing credibility [27].

### Ethical considerations

The study was approved by the Regional Ethical Review Board in Uppsala, Sweden (reg. No.: 2011/07). An informative note regarding the study, directed to all staff, and patients and their families was posted on general noticeboards around the ED. The nursing students and their preceptors received both written and verbal information regarding the purpose of the study. The students were informed that participation was voluntary and that they could withdraw from the study at any time without their education being affected. None of the students were in any dependency position on the researcher who had invited them to participate. The students were assured that the data would be treated confidentially and would be presented in a way that ensured that the participants in the study would remain anonymous. In addition, the patients and relatives were asked for their consent before researchers followed the nursing students at the ED.

## Results

The analysis resulted in an overarching theme: “*Learning about professional identities with respect to situated power*”. This theme illustrates the tensions involved in the mundane ways in which the students were socialized into practical skills in the nursing profession. The theme further highlights how the nurses who precepted them legitimized the ways in which they performed practical skills.

**Fig. 1:**
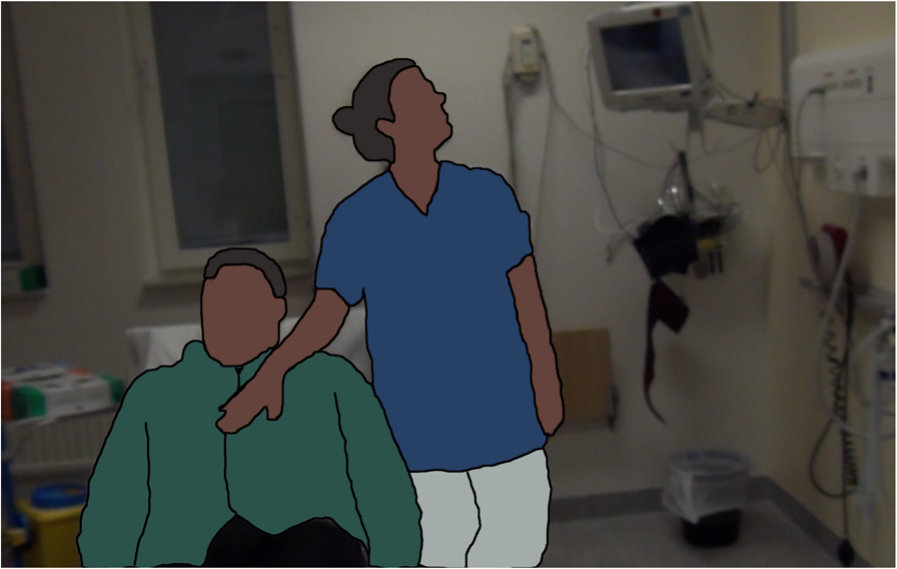
A student nurse checks a patient’s respiratory rate in a non-patient-centred manner

The overarching theme consists of three sub-themes: “*Embodied knowledge*”, “*Divergent ways of assessing and evaluating knowledge*”, and “*Balancing approaches*”*.* Each sub-theme, presented under separate sub-headings, is illustrated with the help of quotations, and examples from the data (see Fig. 1, Tables 1 and 2).

**Table 1:** Example of a mismatch situation: handling a complex procedure

In this case, a blood sample needs to be taken and a PVC is inserted. The patient says, “*It’s difficult to puncture my blood vessels.*” One nurse tries and fails, as does a second nurse. During the procedure much of what is happening is erroneous, resulting in stasis, and leading in turn to the patient’s hand bruising dark blue. The nurse taps quite hard on the patient’s hand although the patient says repeatedly, “*That hurts!*” The nurse, who is wearing gloves with cut fingers, does not disinfect the patient’s skin. The needle goes in and out of the plastic catheter. The patient repeatedly expresses discomfort. The observing student looks pensive. He glances towards the researcher in the room on several occasions (the latter has a nursing/teaching background). A more experienced nurse now enters the room and tries to insert the PVC. The same erroneous performance is repeated, except that this nurse does not have any gloves on. The experienced nurse looks towards the researcher and says, “*It gets this way sometimes.*” There is no discussion with the student afterwards about what she has seen and the student does not ask any questions either.

**Table 2:** Example of when a student takes an independent decision

The student and the preceptor are in the drug room where the student prepares two intravenous injections. The student works independently, with the preceptor observing in the background. The student lifts up the syringes with their contents. “*Great job*,” says the preceptor. “*Put the syringes in your breast pocket and then we can go to the patient*,” he says, pointing at the student’s breast pocket where several pens can be seen sticking out. The student does not say anything, but pauses for a few seconds. Without a comment, he takes a tray and places the syringes on it. The preceptor says nothing.	

### Embodied knowledge

The students stressed the importance of getting “hands-on experience” to complement their previous knowledge. They expressed the need to test their performance in real-life situations and perform their tasks fluently. As one student said:

“*I must practise, practise and practise again. You have to get the feeling in your hands and it is only when I understand the consequences of my performance that I can learn. Otherwise it is impossible to remember how to do it.*”

Several students expressed a preference for first observing how their preceptor performed a specific skill, and checking whether she/he used “tips and tricks” to made the task easier. For example, one student said: “*The nurses may have some small tricks that we can’t read about in a book. If they look good to me, I might try them, and if they don’t work then I might reject them.*” When it was time to perform a task themselves, students felt that it was important to be allowed to perform it independently, with the preceptor as a backup support in the background. The preceptor taking over at this juncture was perceived negatively.

Both the fieldwork and the interview data highlight that some practical skills posed greater challenges for the students compared with other skills, and that the students spent a lot of effort managing these, to become confident in performing them. This was especially the case with inserting a peripheral venous catheter (PVC) and taking blood samples. Several students expressed issues such as: “*Once your hands can do it more automatically, you are able to focus more on the patient and not only on the blood vessels.*” The analysis revealed that the students were often disappointed in their own performance and that they were more nervous at a subsequent performance of a specific skill if they had failed it previously or if they were unable to perform it fluently. Some students explicitly raised issues about their performance failures with their preceptors; others did not. The data also revealed that some students avoided practising a skill that they had not succeeded in previously.

### Divergent ways of assessing and evaluating knowledge

The trust the students had in their preceptor as a “source of knowledge” differed depending on how they valued and reflected on the preceptor’s way of performing practical skills and the preceptor’s ability to explain a patient’s holistic situation. Some of the students expressed total trust in their preceptor as a knowledge source. They admired their preceptor’s knowledge and the way the preceptor performed practical skills even if the observation data sometimes revealed shortcomings. As one student commented, “*You take for granted that they have knowledge and can do what they do.*”

However, some students said that they had sometimes seen a task performed or heard an explanation that they thought differed from what they had learnt at university. Such incidents raised doubts concerning what was actually recommended and what was correct. They based their concerns on their experiences from the university CSL. However, some students added that they understood that certain procedures could be done in different ways as long as the patients’ safety was not compromised. The data also revealed that some students were ill prepared for the work and lacked knowledge, which they seemed unaware of. They performed procedures improperly, sometimes in a way that could harm the patient. Despite this, they valued their preceptor’s way of performing practical skills. Furthermore, some students did not build their performance on the current literature and the way practical skills were taught at the CSL. Even if their performance did not constitute a risk for patient safety, this could be problematic.

Figure 1 illustrates the gap between the technically oriented nature of the clinical practice guidelines and the performance of practical skills as a complex action where the patient would be in the centre. Although patient-centred care is central in nursing education, in Fig. 1 the student is standing behind the patient, and focusing only on the monitor to assess respiratory rate. This position does not allow her to have eye contact with, nor does it enable observation of, the patient, regarding both vital parameters and the patients’ wellbeing (for example, checking for signs of anxiety).

The students also compared different nurses’ ways of handling situations and performing practical skills. They expressed an admiration for nurses who they felt were skilled. One student said, “*My preceptor can always put in a PVC, she does it just as it says in our literature, and she is also very kind to the patients. I hope I can be like her.*” Such accounts illustrate the value that nursing students placed on their socialization into practical skills in situ in the clinical settings.

### Balancing approaches

The analysis of the two data-sets (the fieldwork data and the narrative interview data) revealed that sometimes students were in situations where the nurses who precepted them did not perform a practical skill according to current nursing recommendations and, hence, to the way the students had been taught to perform the same skill at the university CSL. In some of these cases the preceptor acted as if the performance was completely correct; furthermore, the preceptors concerned did not discuss their divergent, and sometimes incorrect, performance with the students (see Table 1).

The students behaved in different ways when faced with mismatch situations of the type illustrated in Table 1. Some students did not raise issues about what they saw although their facial expressions could be interpreted as displaying a negative response (and they later discussed the situation with the researcher in the field). Some students reported that they had really wanted to question what they saw, but had refrained, even when they felt uncomfortable about this decision. They explained that they had not discussed such issues with their preceptors because they did not want to sound critical or cause trouble. As one student put it: “*As an inexperienced student I wouldn’t comment on what they do.*” When describing situations in which current guidelines had not been followed, students sometimes defended the preceptor’s actions. For example, one student said: “*They do not disinfect their hands as they should, but they’ve got a lot to do so it’s not so easy for them to do it.*”

Other students reported that they had asked their preceptor directly if there was a procedure that should be carried out differently, even though some of the details that they described did not affect the patients’ safety. They had made comments like: “*In school we did this procedure in this way.*” The preceptors had varied in their responses to such comments. Some preceptors had had a discussion with the student and had asked them more about what they had learnt at the university. Some students reported that their preceptor had approved of the new information that the student provided; this was perceived positively by students. The preceptors also occasionally took the initiative to read national guidelines together to illuminate their discussion. On the other hand, sometimes a preceptor would tell the student: “*I know that you have learned to do this in another way but I will carry it out in my way.*”

The data also revealed that some students did not perform a skill in an improper manner even though their preceptor had prompted them to do so (see Table 2).

The example presented in Table 2 can be interpreted to indicate that the student knew current recommendations, and that he also had the courage to take his own decision regarding how to perform a practical skill.

## Discussion

An overarching theme regarding tensions in the nursing students’ socialization into clinical practice and their transfer of experiences from the CSL to clinical contexts relates to situated power. The results indicate that the nursing students took different approaches to handling identified mismatch situations, i.e. situations when a practical skill was carried out differently from how it was described in the literature. This reveals different ways in which students navigate tensions related to power differentials. This can be seen in the study’s results, underpinning the sub-themes: “*Divergent ways of assessing and evaluating knowledge*”, and “*Balancing approaches*”. Some students did not comment or ask questions even if a situation included practices that the students themselves assessed as being far from evidence-based. Both in informal conversations and in the interviews, some students expressed regret, saying that they should have said something but had chosen to keep quiet, even if it made them feel ashamed of themselves. This result is in line with Lùanaigh et al. [14] and Monrouxe et al. [28] who report that students felt uncomfortable when they witnessed poor practice, both when they intervened or commented and when they remained silent. However, the students in the present study displayed the ability to distinguish between good and poor practices and this is in line with the findings of previous studies [14, 28]. Our interpretation about why some students choose to not speak up relates to the hierarchy in clinical practice. Students recognize their position as newcomers and are afraid of being reprimanded; this is related to situated power. They also want to gain the acceptance of the group, which is easier if they “fit in” and adopt the practices advocated by their preceptor. Furthermore, previous research suggests that students take the side of the group they currently belong to [29], which may explain why students in the present study sometimes defended or excused their preceptors’ incorrect performance. On the other hand, some students spoke up when they identified a mismatch situation, for example by asking their preceptor for the reason behind a certain practice, or commenting that they had learnt it in a different manner at the university. Most students, in their own performance of skills, avoided practices that differed from what they had learnt, even when their preceptor had proposed alternative methods. The analysis suggests that it was both the students’ personal knowledge and degree of self-confidence, and the interaction between the student and the preceptor that shaped the students’ approach. According to Bickhoff et al. [30], it is difficult for students to question poor practice or to decline to do something in a certain way when this is required of them. Going against the grain takes moral courage. Moral courage, according to Lachman [31], involves bridging the gap between personal knowledge and values, and the obligations of a profession. Personal knowledge and experiences can increase students’ self-confidence and this might help them to develop moral courage. One way to increase students’ self-confidence is related to the sub-theme *Embodied knowledge.* Gaining a “hands-on feeling” allowed students to be more confident in the fluency of their own performance in a patient situation. The need for fluency and confidence can be understood in terms of the students realizing that nurses who are skilled at their work and manage practical skills in a clinical setting can experience their work as rewarding [6]. In line with previous research [32, 33], students in our study expressed that they often felt stressed and worried that their performance might cause injury to their patients. Thus, becoming fluent in their performance has the potential to strengthen students’ self-confidence. Furthermore, students can more easily use their knowledge and adopt new knowledge and skills when they feel confident.

The students used different strategies to get a “hands-on feeling”. Several said that they first wanted to see if the preceptor had some “tricks” they could use and that they had the preceptor explain these. Above all, they wanted the ability to perform different procedures repeatedly on their own, with the preceptor acting as a guide and supervisor. This is in line with previous descriptions in the literature about how important practice time in clinical settings is, and also how important the preceptor’s approach is [15, 16]. Reflecting on actions has previously been described as a prerequisite for developing and learning practical skills [34]. This highlights the importance of both university educators and the preceptor’s role for socializing students in this process.

In this study, there were some students who displayed a lack of proficiency in skill performance. They performed skills improperly and seemed unaware of this. Sometimes potential risks to the patients were evident when task performance was erroneous. Puncture of the veins and inserting a PVC were tasks that the students described as the most challenging, which is in line with Marshburn et al. [35]. Ravik et al. [23] describe some participants who even performed some PVC steps incorrectly in the CSL and transferred these mistakes to the clinical setting. However, what is taught in a lecture in the CSL does not automatically lead to personal learning or the ability to transfer what has been taught [22]. Students have different backgrounds and their learning and development is shaped by their previous experiences, interpersonal communication and interaction with others in a community of practice [19, 20]. Another explanation regarding difficulties related to transfer of knowledge between these different contexts may be related to students’ learning approach in the CSL. Learning in the CSL is based on a mechanical approach with a focus on repetition and familiar elements, an approach that may itself hamper some students’ ability to transfer knowledge to another, much more complex context [23]. To be prepared for performance of practical skills in a real patient situation also requires that students take responsibility on their own to practise their own performance with a reflective approach in the CSL, before they enter clinical settings. Failing this, both their ability to reflect on their own performance of practical skills in clinical settings and their possibility to develop an embodied knowledge, based on the literature, are hampered [1, 32].

### Methodological considerations

Researchers’ preconceptions and prior contacts with persons in the field may possibly have affected the researchers’ observations and analysis. However, three of the four researchers who have authored this study (ME, SBG and KB) have a range of experiences significant for this study. The fourth researcher (RA), who did not participate in the fieldwork, contributed in the analysis. This diversity of experiences provided a strong background for conducting fieldwork from different perspectives and enabled richer, analytically driven discussions of the culture of clinical practices.

## Conclusion

Nursing students do not automatically possess the ability to transfer knowledge from one setting to another. Even if they have been taught how to perform a practical skill, their development is shaped by their experiences and interactions with others in a real patient situation. Nursing students’ transfer of acquired knowledge to the clinical context relates to situated power. Students use different ways to navigate the tensions that emerge when practical skills are carried out differently compared with the way they have been taught at the university. Hence, it is important that the preceptors understand how the hierarchy in clinical practice shapes students’ behaviour in clinical settings. Nursing education and preceptors have a mutual responsibility to facilitate students’ increased self-confidence. Reflecting together on actions is a prerequisite for learning and developing practical skills and, thus, professional identities. This highlights the importance of the role of both university educators and preceptors in socializing students in this process.

## Abbreviations

CSL: Clinical skills laboratory
ED: Emergency department
PVC: Peripheral venous catheter
